# [Retracted] Effects of miR‑138‑5p and miR‑204‑5p on the migration and proliferation of gastric cancer cells by targeting EGFR

**DOI:** 10.3892/or.2023.8677

**Published:** 2023-12-05

**Authors:** Yi Wang, Haiyang Zhang, Shaohua Ge, Qian Fan, Likun Zhou, Hongli Li, Ming Bai, Tao Ning, Rui Liu, Xia Wang, Ting Deng, Le Zhang, Guoguang Ying, Yi Ba

Oncol Rep 39: 2624–2634, 2018; DOI: 10.3892/or.2018.6389

Following the publication of the above paper, it was drawn to the Editor's attention by a concerned reader that the numerous immunohistochemical images shown in Fig. 1D on p. 2626 exhibited a number of overlaps comparing among the data panels, such that data which were intended to show the results from differently performed experiments were likely to have been derived from a smaller number of original sources. A subsequent independent investigation of the data in this paper in the Editorial Office also revealed that certain of the cell migration and invasion assay data shown in Fig. 4A on p. 2629 were strikingly similar to data that had previously appeared in a couple of already published papers written by different authors at different research institutes.

Owing to the fact that the contentious data in Fig. 4 in the above article had already been published prior to its submission to *Oncology Reports*, in addition to the matter of several panels in Fig. 1D showing overlapping data, the Editor has decided that this paper should be retracted from the Journal. The authors were asked for an explanation to account for these concerns, but the Editorial Office did not receive a reply. The Editor apologizes to the readership for any inconvenience caused.

